# Supplementary material for: Complex‐centric proteome profiling by SEC‐SWATH‐MS
Source: Mol Syst Biol. 2019 Jan 14;15(1):e8438. doi: 10.15252/msb.20188438 (PMC6346213; doi:10.15252/msb.20188438)
Supplement: Supplementary file 7 — Dataset EV6 [file MSB-15-e8438-s007.zip › feature_plots_bioplex/Q9UIV1.pdf]

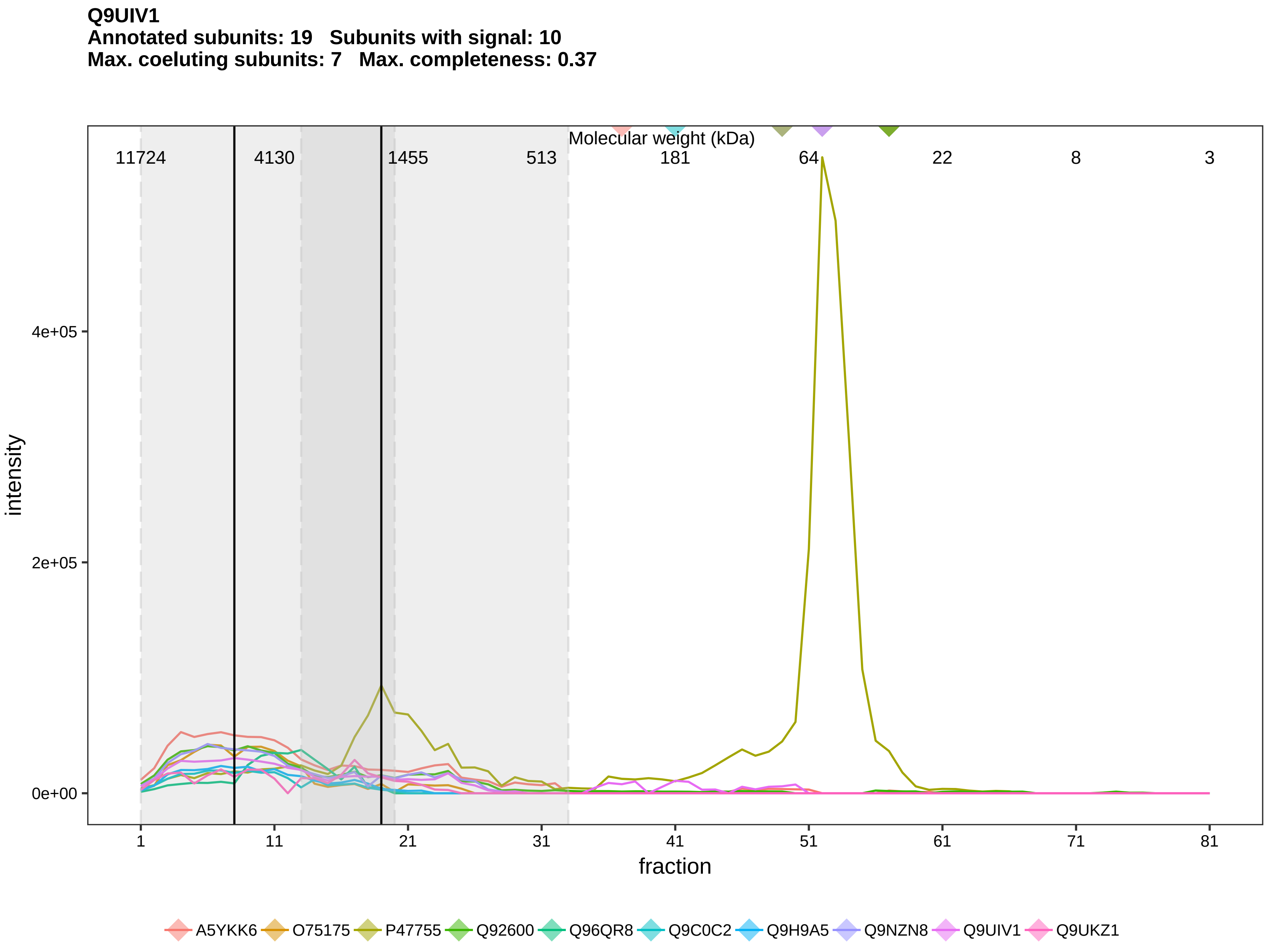
Q9UIV1
Annotated subunits: 19   Subunits with signal: 10
Max. coeluting subunits: 7   Max. completeness: 0.37
Molecular weight (kDa)
11724
4130
1455
513
181
64
22
8
3
intensity
4e+05
2e+05
0e+00
1
11
21
31
41
51
61
71
81
fraction
A5YKK6
O75175
P47755
Q92600
Q96QR8
Q9C0C2
Q9H9A5
Q9NZN8
Q9UIV1
Q9UKZ1
